# Influence of Socioeconomic Status on Knowledge of Obesity and Diabetes among Adolescents in Chennai, South India

**DOI:** 10.3390/children4080061

**Published:** 2017-07-25

**Authors:** Pranati Panuganti, T. S. Mehreen, Ranjit Mohan Anjana, Viswanathan Mohan, E. Mayer-Davis, Harish Ranjani

**Affiliations:** 1 Department of Nutritional Epidemiology, Gillings School of Global Public Health, University of North Carolina at Chapel Hill, Chapel Hill, NC 27599, USA; ppanug@gmail.com (P.P.); mayerdav@email.unc.edu (E.M.-D.); 2 Madras Diabetes Research Foundation & Dr. Mohan’s Diabetes Specialities Centre, WHO Collaborating Centre for Non-Communicable Diseases Prevention and Control, ICMR Centre for Advanced Research on Diabetes, 4, Conran Smith Road, Gopalapuram, Chennai 600086, India; mehreen.begum@gmail.com (T.S.M.); dranjana@drmohans.com (R.M.A.); drmohans@diabetes.ind.in (V.M.)

**Keywords:** diabetes, prevention, awareness, intervention, school

## Abstract

The Obesity Reduction and Awareness of Non-communicable disease through Group Education (ORANGE) Phase II program, is a school-based intervention aimed at healthy lifestyle practices for sixth and seventh grade adolescents (*n* = 2345) attending private (*n* = 1811) and government (*n* = 534) schools in Chennai. The objectives of this paper are (a) to describe the intervention activities and their outcomes qualitatively and (b) to report changes in body mass index (BMI) of the intervention group participants. This intervention strategy used a teacher-peer-training model in each school for long-term sustainability of the lessons learned from this program. During each intervention session, teachers led a classroom discussion on the health topic of interest, and peers facilitated small-group learning activities. Anthropometric measurements of participants were assessed pre- and post-intervention. We found government school students perceived hygienic actions (e.g., drinking clean water, taking baths daily) as healthy habits for preventing diabetes, whereas private school students associated an expensive lifestyle (e.g., eating at restaurants, riding a car) with diabetes prevention. Overall, the mean post-intervention BMI (18.3 kg/m^2^) was in the normal range compared to the pre-intervention BMI (17.7 kg/m^2^) (*p* < 0.0001). These results suggest that future interventions should be tailored for adolescents from different socio-economic groups while acknowledging their varied perceptions.

## 1. Introduction

Recent estimates from the International Diabetes Federation (IDF) reported that India has 69.2 million people with diabetes and this number is predicted to surpass 123.5 million by the year 2040 [1]. This rapid escalation of diabetes prevalence in India has been attributed to several factors, including rapid urbanization, growth of the middle class, sedentary lifestyle, and diets rich in carbohydrates and calories but low in fiber. These unhealthy patterns in diet and lifestyle in India are influenced by social attitudes and cultural practices [2,3,4]. Over the past 20 years, there has also been an emerging occurrence of type 2 diabetes among children and adolescents, which parallels the increasing prevalence of obesity in this age group [5]. In Chennai, Tamil Nadu, the prevalence of obesity among schoolchildren was found to be 21.4% in private schools (higher socioeconomic status) and 3.6% in government schools (lower socioeconomic status) [6].

Despite India’s large diabetes burden, over 50% of individuals with diabetes in this subcontinent are unaware of their condition [7]. In 2001, Norris et al. [8] have shown that educating people at-risk for diabetes is an effective measure for preventing or managing the disease. The World Health Organization (2009) [9] has also found such population-based interventions to be helpful, cost-effective strategies in preventing at least 80% of non-communicable diseases, such as type 2 diabetes in low- and middle-income countries [10].

Lifestyle interventions, which are shown to reduce the risk of type 2 diabetes in adults, have also been recommended for youth [11,12]. To tackle childhood obesity and prevent diabetes, recent literature calls for comprehensive evidence-based strategies, which are both practical and cost-effective [13]. Furthermore, other studies emphasize the importance of culturally relevant and community-based programs, which directly engage participants in the intervention process [14,15].

This study was part of a large school-based program in Chennai, South India called Obesity Reduction and Awareness of Non-Communicable diseases through Group Education (ORANGE). In Phase I of the ORANGE study, 18,955 children and adolescents across 51 government and private schools in Chennai, and 1519 children and adolescents in Chennai colonies were screened for obesity, diabetes, high blood pressure, and other risk factors for non-communicable diseases (NCDs). Participants, teachers, and parents were also given a self-administered questionnaire to assess demographics, knowledge about NCDs, and lifestyle practices including diet and physical activity [6,16].

The aim of Phase II of the ORANGE study was to implement a school-based co-curriculum intervention for diabetes awareness, preventative methods, and self-management in adolescents across select private and government schools in Chennai. This intervention strategy used a teacher-peer-training model in each school for long-term sustainability of the lessons learned from this program. During each intervention session, teachers led a classroom discussion on the health topic of interest, and peers facilitated small-group learning activities.

Most prior studies on diabetes education interventions were limited to analyzing changes in biochemical markers or anthropometric measurements, such as glucose tolerance, blood pressure, and body mass index (BMI). In addition to collecting these anthropometrical measurements, this study also analyzed and evaluated themes emerging from the participant’s qualitative responses to the learning activities. The two-fold objective of this paper is (a) to describe the intervention activities and their outcomes qualitatively and (b) report changes in body mass index of the intervention group participants.

## 2. Materials and Methods

### 2.1. Participants

ORANGE Phase II is a 1-year, school-based co-curriculum intervention for diabetes awareness, prevention and self-management in children and adolescents across Chennai. The program was implemented in seven private and three government schools across Chennai, purposively selected to represent different socioeconomic groups and geographic localities in Chennai. Students of middle to upper socioeconomic background tend to attend private schools, while those from lower socioeconomic backgrounds usually go to government schools. All sixth and seventh grade students attending these chosen schools were eligible to participate in the intervention program. During this period, pubertal and/or related behavioral changes can further result in a decline in physical activity and adoption of unhealthy dietary habits, which can be directly linked to type 2 diabetes [17]. Hence, this age group was chosen to help the teenagers adopt healthy behavioral changes before their lifestyle habits were permanently established.

This study protocol was approved by the institutional ethics committee of the Madras Diabetes Research Foundation (MDRF) with Registration No. ECR/194/Inst./TN/2013 and the trial is also registered in the Clinical Trials Registry-India (CTRI) with ID number CTRI/2016/09/007323.

### 2.2. Structure

**Figure 1:**
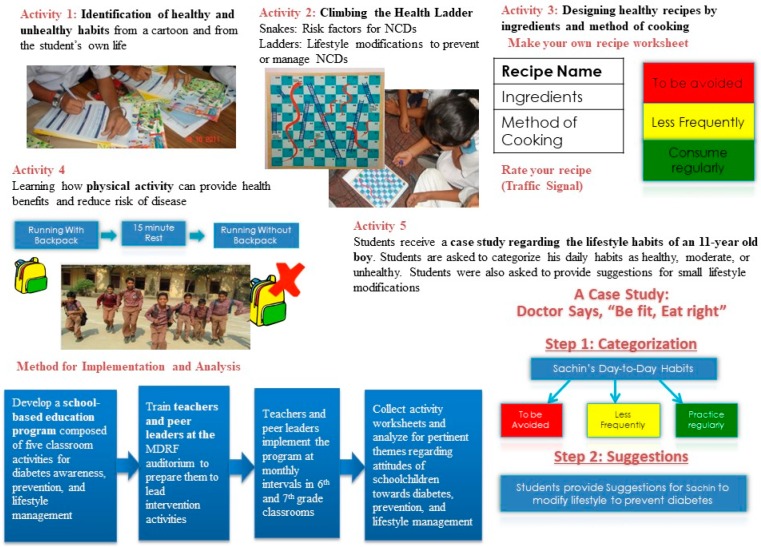
Description of classroom intervention activities, conceptualized, designed and developed into an activity manual by Health-Related Information Dissemination Amongst Youth (HRIDAY), New Delhi in partnership with Arogya World Inc., Chicago and Bengaluru, and the Madras Diabetes Research Foundation (MDRF), Chennai. NCDs: non-communicable diseases.

The intervention was composed of five classroom activities, delivered at monthly intervals in each school. Each activity provided background information about a certain health topic of interest. The general goal of each activity was to sensitize participants to diabetes awareness and prevention. The activities were conceptualized, designed and developed into an activity manual by Health-Related Information Dissemination Amongst Youth (HRIDAY), New Delhi in partnership with Arogya World Inc., Chicago and Bengaluru and the Madras Diabetes Research Foundation (MDRF), Chennai. The medium of instruction used for delivering the intervention program was English and Tamil at private and government schools, respectively (Figure 1). Detailed activity descriptions have been provided in Appendix A.

Teachers and peer leaders are an essential component to the structure of this intervention program. Teachers were the natural choice to lead each classroom activity, due to their role in influencing their students’ attitudes and behavior positively. One student from each class was also selected as a peer leader by the teacher coordinator, due to good leadership qualities and amiability among classmates. Peer leaders facilitated small group discussions, led activities, and acted as messengers between students and teachers. Training was held at the MDRF auditorium to train and prepare both teacher coordinators and peer leaders to lead the intervention activities. Teachers and peer leaders are important players in this program because they already maintain social or authoritative ties with the students. Moreover, in this age group hearing information from peers may be more effective than hearing it from adults [18].

### 2.3. Anthropometric Measures

Height was measured in centimeters using a stadiometer. Subjects were asked to stand upright without shoes with their back against the scale, heels together, and eyes directed forward. With the help of the headrest, height was measured to the nearest centimeter. Weight was measured to the nearest 0.1 kg using a standard weighing scale that was kept on a firm horizontal surface. The scale was checked every week, and calibration was done with known weight. The BMI was calculated using the formula of weight (kg)/height (m^2^).

### 2.4. Data Analysis

The student responses to activity worksheets and pre-post BMI primarily served as the collected data for this intervention, and were analyzed using SAS 9.4 (Cary, NC, USA). Participant characteristics including age, gender, school type (private or government), class (sixth or seventh grade), and intervention compliance were described overall and by school type and gender. Intervention compliance was defined as ‘compliant’ (completed 4–5 activities total) and ‘non-compliant’ (completed <4 activities total). The pre- and post-intervention BMI of participants were also described overall and by school type, gender, and intervention compliance status. Numbers and percentages were tabulated for students completing each activity, different student responses, and correctness and categorization of responses. Independent sample *t*-tests, paired sample *t*-tests and Chi-square test of proportions were performed to assess differences in responses and anthropometric measurements between government and private school students split by gender and age group as well pre-post intervention. Finally, emergent themes among student responses to intervention activities were identified by citing common responses to intervention activities, stratifying the responses by school type, and drawing thematic conclusions from the individual student responses and relevant published literature. No measurable data was collected or analyzed for Activities 2 and 4, which involved educational games that taught school children about diabetes and its prevention, rather than assessing their knowledge.

## 3. Results

**Figure 2:**
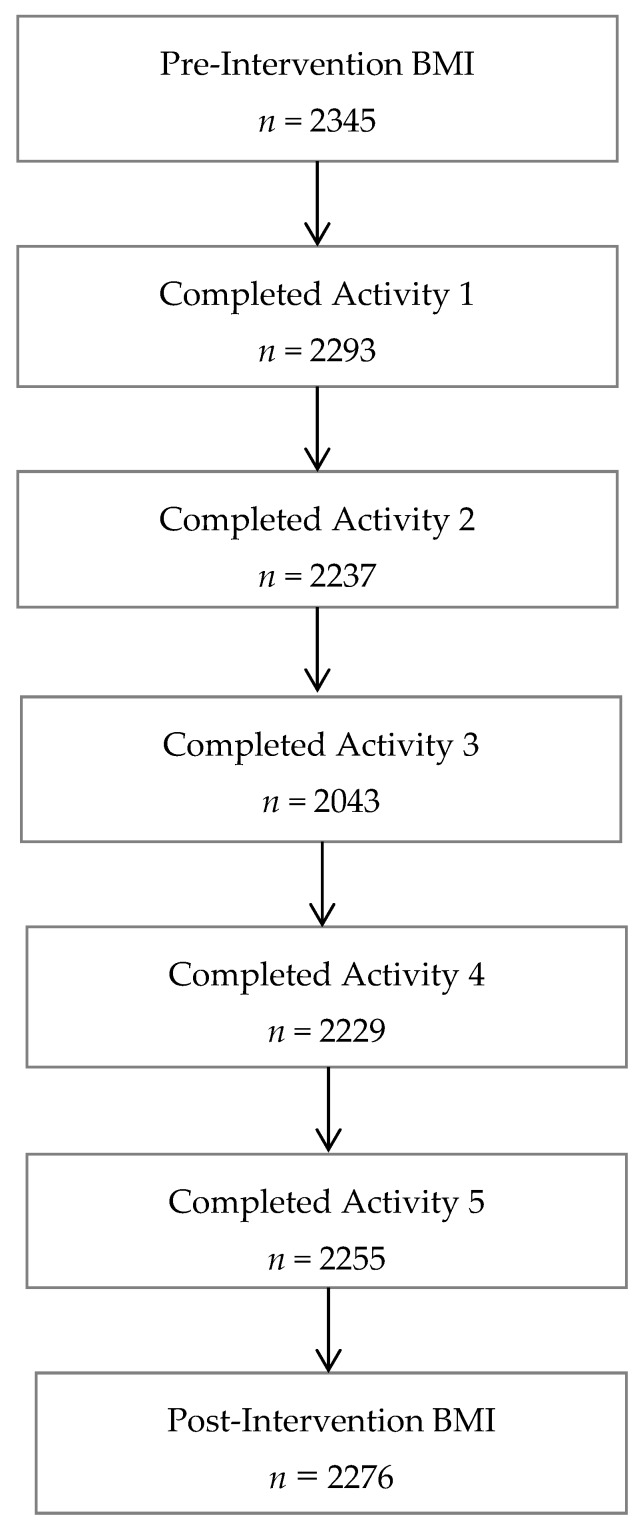
Flowchart of student participation in the ORANGE Phase-II school intervention.

Overall, 2345 students were enrolled in the ORANGE Phase II intervention program. Out of the total student population, 2293 (97.8%) students participated in Activity 1, 2237 (95.4%) in Activity 2, 2043 (87.1%) in Activity 3, 2229 (95.1%) in Activity 4, and 2255 (96.2%) in Activity 5. We limited our final sample to 2276 students who attended both the pre- and post-intervention assessments (Figure 2). Of these 2276 students, 95.8% completed 4–5 intervention activities (defined as “compliant”), while 4.2% completed less than 4 activities (defined as “noncompliant”). Furthermore, a greater proportion of private school students (98%) as compared to government school students (88%) were compliant (Table 1).

### 3.1. Participant Characteristics

Of the 2276 total participants, a greater proportion attended private schools (*n* = 78.4%) than government schools (*n* = 21.6%) (*p* < 0.001). There was a lower proportion of female participants (43.2%) overall, and this gender bias was larger in government schools. In both government and private schools, the mean age of the students was 11.1 years and there were roughly equal numbers of students from the sixth grade (50.9%) and the seventh grade (49.1%). These results are displayed in Table 1.

### 3.2. Pre- and Post- Intervention BMI

Overall, the mean post-intervention BMI of 18.3 kg/m^2^ was greater than the mean pre-intervention BMI of 17.7 kg/m^2^ (*p* < 0.0001), although both of these values are within the BMI cut-points for normal weight children in India (Table 2). The mean BMI of study participants improved across school type (government: 0.7 kg/m^2^ and private: 0.5 kg/m^2^ increase) and gender (boys and girls: 0.6 kg/m^2^ increase) resulting in an increase in the number of students with normal weight. The percentage of overweight and obesity in adolescents was 16.2% and there was no significant change in the proportion of overweight and obesity in adolescents after intervention. Nearly 32% of the children were malnourished, which reduced to 25% post intervention (*p* < 0.0001). In government school students, the proportion was higher (56.5%) and decreased to 47.4% post intervention.

### 3.3. Activity Responses and Feedback from Peer Leaders and Teachers

#### 3.3.1. Activity 1: Way to Health

In Activity 1, students were taught about the healthfulness of various lifestyle habits, and asked to identify habits from their life and from a picture that they perceived to be healthy and unhealthy.

Many private school students perceived eating at restaurants to be a healthy habit, while government school students considered respecting elders or studying to be habits contributing to good health rather than good behavior. Of all perceived unhealthy habits, a few were healthy habits (running, playing, or eating fruits) or those that can be categorized as good habits which do not necessarily contribute to reducing the risk of obesity and diabetes (cleaning, helping parents, etc.) Many students in government schools also identified at least one habit associated with being unhygienic or bad behavior as an unhealthy habit. These include “wearing untidy dress” or “hitting others”.

**Figure 3:**
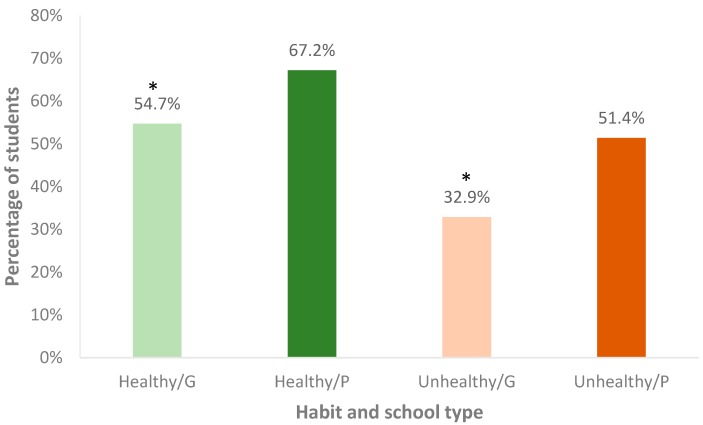
Percentage of students correctly identifying all five healthy habits from a picture in private (P) and government (G) schools. * Difference between P and G is statistically significant at *p* < 0.001.

In both private and government schools, students were able to identify all five healthy habits better than the five unhealthy habits from the picture shown to them. However, a significantly higher percentage of students in private schools was able to correctly identify all five healthy habits (67.2%) and unhealthy habits (51.4%) as compared to students in government schools (54.7% and 32.9%, respectively, *p* < 0.001) (Figure 3).

In private schools, the peer leaders for Activity 1 claimed the activity was “simple, useful, and enjoyable” and helped them to gain “knowledge about diabetes and obesity” while “identifying healthy and unhealthy habits”. Private school teachers suggested the “pictorial handouts helped to gain more awareness”. Government school peer leaders also noticed that students enjoyed “learning from the picture” and “learning about healthy and unhealthy habits”.

#### 3.3.2. Activity 2: Climbing the Health Ladder

In the game of snakes and ladders, peer leaders liked “how healthy habits represented going up ladders and unhealthy habits were represented by snakes” and mentioned that “students participated enthusiastically”. They also enjoyed active participation in the game, as one peer leader claims, “Instead of talking hours of theory, this organization has represented [the information] in a way that makes us easy to understand”. Teachers also agree that “with [snakes and ladders as a] known game, [it] imparted the best knowledge about diabetes in a simple and enjoyable way”. Logistics-wise, teachers suggested that “materials for the game could have been provided, because school has to take print outs and procure other miscellaneous items”.

#### 3.3.3. Activity 3: Pass It on-The Traffic Signal

Activity 3 required students to design and grade their own recipes using the traffic light colors: red (avoid completely or eat in very limited quantities), yellow (eat in moderation) or green (eat regularly). The selection of zones is based on the ingredients and method of cooking. In government and private schools, most students designed recipes they perceived to be green. Common green recipes that students designed included fruit salad and vegetable sandwiches. Yellow recipes that were popular among students included dosa, cheese sandwiches, and rice dishes such as lemon rice and tomato rice. The few red recipes that students designed included fried rice, biryani, and noodles.

**Figure 4:**
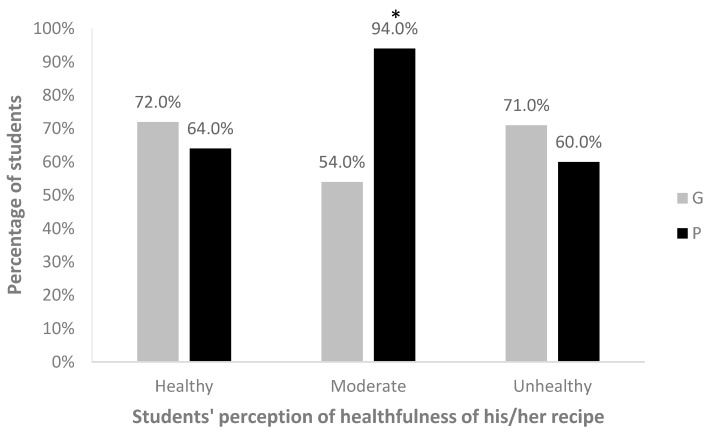
Percentage of students correct in grading the healthfulness of their own recipe, as determined by a dietician, in private (P) and government (G) schools. * Difference between P and G is statistically significant at *p* < 0.001.

When a dietician graded these recipes, there was no significant difference in the proportions of private and government school students (64% and 72%, respectively) who were correct in grading their recipe as green. An equal proportion of both government and private school students (36.4% and 37.2%, respectively) designed recipes they perceived to be yellow (*p* = 0.881). In private schools, only 54.2% of students creating yellow recipes were correct in their grading, while 94.2% were correct in government schools (*p* < 0.001) (Figure 4).

Peer leaders for Activity 3 appreciated that “students were allowed to make their own recipe rather than being handed a healthy recipe.” They also said, “going through other recipes and helping them understand about eating healthy foods was good”. Likewise, teachers said “Students enjoyed writing the recipe of their favorite dish and the ingredients used; they understood the importance of healthy options in food and eating a balanced diet”. Teachers also said the “food pyramid was very useful and helped in easy understanding for the students”.

#### 3.3.4. Activity 4: Jump Forward and Race with Pace

In Activity 4, peer leaders said that students “had fun running with bags” where “excess weight was represented as a heavy bag”. They said the activity “helped realize that being obese is a problem/burdensome and all are aiming to be fit and healthy”. Likewise, teachers exclaimed that “Children co-operated and liked the activity very much”, and it “helped understand problems of obesity”.

#### 3.3.5. Activity 5: Doctor Says “Be Fit, Eat Right”

In Activity 5, students were given a case study regarding a young boy named Sachin, and were asked to identify healthy and unhealthy lifestyle practices from Sachin’s day-to-day life, and offer small lifestyle modifications for Sachin.

Students receiving the intervention program classified the character of Sachin’s behaviors from the case study as green (practice regularly), yellow (in moderate), or red (avoid completely). Sample dietary behaviors and their classifications included “Eats home-made tiffin—green” and “Has burger and sweetened beverages everyday—red”. Sample behaviors concerning physical activity or sedentary behavior include “Walks with mom—green”, “Takes car to school—yellow”, and “Avoids games period—red”.

**Figure 5:**
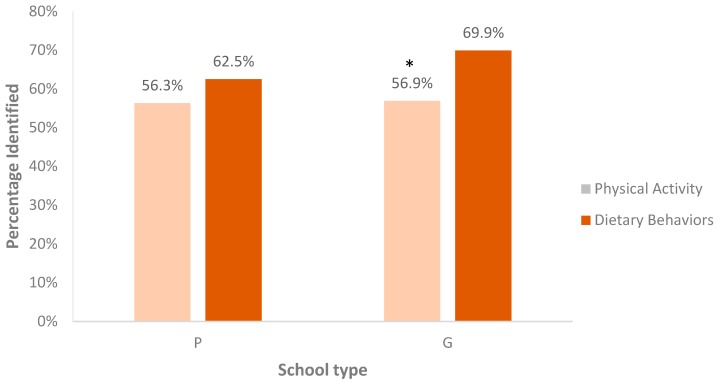
Percentage of “Be Fit” and “Eat Right” activities correctly classified in private (P) and government (G) schools. * Difference between P and G is statistically significant at *p* < 0.001.

Among dietary behaviors, greater proportions were correctly classified as red, yellow, or green by government school students (69.9%) as compared to private school students (62.5%), (*p* < 0.001). These results are displayed in Figure 5. Both males and females correctly classified an equal proportion of physical activities as red, yellow, or green (*p* = 0.6031). However, females correctly classified a significantly greater percentage (67.8%) of dietary behavior as compared to males (63.6%) (*p* < 0.005).

Peer leaders for Activity 5 liked how it “was a summary of all the activities”, as they “found it very useful and understood the importance of eating right along with maintaining a healthy body”. They also liked how “it used an example of an unhealthy boy” who they could relate with, and it “let the student step into the shoes of a doctor and give him suggestions”.

## 4. Discussion

In order to prevent or manage childhood obesity, comprehensive, multidisciplinary, and evidence-based lifestyle interventions are needed. Developing countries such as India require novel strategies at the community level, which engage participants in the intervention process [14]. The most practical and cost-effective setting for educating children and adolescents is the school [13,18]. Other successful school-based health interventions have shown that they encourage short-term lifestyle changes, while at the same time increasing knowledge and attitudes among students [15,19].

This paper essentially reports the results (assessed qualitatively) of our school-based co-curriculum intervention program that aimed to educate children and adolescents about healthy lifestyle practices to prevent or control obesity and diabetes. The ORANGE health education implementation appealed to children and adolescents from select government and private schools in Chennai, the largest metropolis of Southern India. Encouraging participants to adopt healthy lifestyle practices (dietary intakes, increasing physical activity, avoiding sedentary lifestyle and avoiding stress) has been the major recurring message across this five-part intervention.

Although adolescents may be reluctant to make changes related to healthy living [20], timely social influences can positively affect their behavior [19]. Hence, peer leaders from each school were chosen to guide the program. Each lesson succeeded in engaging students themselves to express and analyze their own opinions and beliefs regarding healthy living, as evident in the high response rates and feedback from peer leaders and teachers. Overall, the findings from this study support such an intervention in empowering children and adolescents to reduce risk of diabetes through healthy living rather than medication or only weight loss.

Assessing themes that emerge from the participant responses is important, because this knowledge can be spread to their peers, family, and others in the community whereby relevant interventions can be created [21]. Several themes have emerged from the data (Table 3), which call for simple modifications to such programs in future.

The government school students had difficulty in distinguishing between non-communicable and infectious diseases. The underlying reason is that these students come from low-income families who may be unable to think beyond their immediate needs such as clean water and hygiene. Perhaps for such reasons, government school students struggled to comprehend the need to prevent non-communicable diseases like obesity and diabetes.

In private schools, there was misinterpretation of a costly activity or meal as a healthy habit. We believe that private school students mistakenly associate wealth with a healthy lifestyle, leading them to perceive certain unhealthy behaviors to be healthy. Private school students come from higher income families, who can afford to eat out at restaurants and have access to television sets, video games, and other activities associated with unhealthy eating and physical inactivity [22].

There was a general lack of awareness of physical activity and increased emphasis on dietary behaviors in both government and private school students. Specifically, children were unaware of how the built environment influences both their energy intake and expenditure. Certain barriers to exercise include inadequate places to exercise, or a desire to avoid pollution outdoors [23]. Familial pressure to succeed in school may also cause children to spend more time studying, leading to more sedentary behavior [24,25].

In India, urbanization and industrialization have upset the energy balance equation [26]. With increased mechanization, the individual workload has decreased, while sedentary activities and energy intake have increased. Lack of awareness of the importance of physical activity suggests that both government and private schools are in dire need of more awareness of the importance of increasing physical activity to prevent obesity and type 2 diabetes.

The issues demonstrated in these themes also present the necessity of modified intervention programs tailored for children and adolescents from different socioeconomic classes. An intervention restructured for government school students may aim to address diabetes prevention while acknowledging the basic, life-sustaining needs of lower-income students. On the other hand, an intervention designed for private school students should help students understand that spending extra money may not be always correlated with healthy living.

### 4.1. Strengths

This study of a school-based co-curriculum diabetes education intervention, was guided by the social-cognitive theory, which takes advantage of adolescence and puberty as a critical development period. Most previous school-based health education interventions in India have only assessed weight-related outcomes or type 2 diabetes risk factors. The main strength of our study is the large sample size and delivery of the program through teachers and peer leaders in a group setting. This approach can help in the scaling up and sustainability of such intervention programs. Finally, the reduction in the number of participants who were malnourished post-intervention, more so in government schools, is an encouraging sign.

### 4.2. Limitations

Although we assessed BMI both pre- and post-intervention, it is difficult to utilize BMI as a marker for intervention success, as there are many confounding factors including age, gender, and socioeconomic status. Furthermore, the intervention was carried out over a 1-year time span during which time the child would grow rapidly both in height and weight due to pubertal changes and thus the BMI would change. The changes in height and weight are provided in Table B1 as part of Appendix B. BMI did increase post-intervention (especially in government schools). These changes could be attributed to changes in lifestyle parameters such as physical activity and dietary pattern of adolescents post-intervention. Unfortunately, we do not have much data on physical activity and diet patterns before the intervention to support this conclusion. This is a limitation of the study.

Another limitation is due to resource constraints. At times, instead of the school staff trained on the program delivering the intervention the study, team staff would need to help in completing the activities. 

## 5. Conclusions

This study shows that while planning prevention programs for non-communicable diseases at the school level, there is no ‘one size fits all’ intervention that can be applied to schools across different socioeconomic groups. This school-based co-curriculum program was effective in educating adolescents about healthy lifestyle modifications to prevent diabetes and improve overall health status. However, the themes that have emerged from this qualitative study also indicate the need to adapt the intervention to different types of school settings across India and other developing countries.

### Key Messages

- A school-based co-curriculum program for educating adolescents about healthy lifestyle modifications to prevent diabetes and improve overall health status was well-received in a sample of government and private schools in Chennai, India.
- This study found divergences in comprehension of the diabetes education program between students from government (low socio-economic status (SES)) and private (high SES) schools.
- Intervention strategies must be adapted to the culture and socioeconomic status of students from different school settings.

## Figures and Tables

**Table 1:** Characteristics of the Obesity Reduction and Awareness of Non-communicable disease through Group Education (ORANGE) study participants by school type.

Variables	No. of Participants (%) (*n* = 2276)	School Type		
		Private No. (%) (*n* = 1784)	Government No. (%) (*n* = 492)	*p*-Value
Age in years * (mean age = 11.1)				
10	561 (24.7)	452 (25.3)	109 (22.2)	0.0003
11	1016 (44.6)	820 (46.0)	196 (39.8)	
12	601 (26.4)	447 (25.1)	154 (31.3)	
13	98 (4.3)	65 (3.6)	33 (6.7)	
Gender *				
Boy	1294 (56.9)	944 (52.9)	350 (71.1)	<0.0001
Girl	982 (43.2)	840 (47.1)	142 (28.9)	
Intervention Compliance *				
Compliant	2181 (95.8)	1748 (98.0)	433 (88.0)	<0.0001
Non-compliant	95 (4.2)	36 (2.0)	59 (12.0)	
Anthropometric measures ^†^ (mean ± SD)				
Height (cm)	147.0 ± 8.5	148.5 ± 8.0	141.3 ± 7.5	<0.0001
Weight (kg)	39.9 ± 10.9	41.7 ± 10.7	33.5 ± 9.1	

* Difference in characteristics across school type were obtained by chi-squared test. ^†^ Difference in anthropometric measures across school type were obtained by independent sample *t*-test. SD: Standard Deviation.

**Table 2:** Pre-post body mass index of ORANGE study participants, overall and by school type and gender.

	Pre-Intervention BMI		Post-Intervention BMI		*p*-Value *
	*n*	Mean ± SD	*n*	Mean ± SD	
Overall	2276	17.7 ± 3.7	2276	18.3 ± 3.8	<0.0001
School					
Government	492	15.9 ± 3.1	492	16.6 ± 3.4	<0.0001
Private	1784	18.2 ± 3.7	1784	18.7 ± 3.8	<0.0001
Gender					
Boy	1294	17.4 ± 3.6	1294	18.0 ± 3.8	<0.0001
Girl	982	18.0 ± 3.7	982	18.6 ± 3.8	<0.0001

* Difference in pre- and post-intervention. BMI was obtained by paired sample *t*-test. BMI: Body Mass Index.

**Table 3:** Emergent themes from the ORANGE Phase II intervention activities.

Theme	Common Outcomes	Rationale
Difficulty in distinguishing between a non-communicable and infectious disease among government school students	Misinterpretation of unsanitary, irresponsible, or irrespective habits as lifestyle behaviors causing diabetes.	Government school students often come from low-income families, who may struggle to think past their immediate needs (such as clean water and hygiene), and better comprehend the message of the intervention program.
Misinterpretation of a costly activity or meal as a healthy habit among private school students	Private school students often suggest eating at restaurants and riding cars as healthy lifestyle modifications to prevent diabetes. They may mistakenly associate wealth with a healthy lifestyle, and perceive certain unhealthy behaviors to be healthy.	Private school students come from higher income families, who can afford high-calorie restaurant food, television sets, video games, and other pleasures correlated with unhealthy eating and physical inactivity [22].
Lack of awareness of physical activity and increased emphasis on dietary behaviors among both private and government school students	When asked to identify unhealthy lifestyle behaviors related to diabetes, more students correctly listed improper eating habits rather than physical inactivity or sedentary behaviors. Also, many students listed studying as a healthy lifestyle behavior to help prevent diabetes, even though it is a sedentary behavior.	It appears that children are unaware of how the built environment influences both their energy intake and energy expenditure. Certain barriers to exercise include inadequate places to exercise, or a desire to avoid pollution outdoors [23]. Familial pressure to succeed in school may also cause children to disregard studying as a sedentary behavior [24,25].

## Appendix A

### Appendix A.1. Activity 1

The objective of the first activity, *Way to Health*, was to educate students about various healthy and unhealthy lifestyle practices, and to help students classify the healthfulness of their own lifestyle practices. The major theme from this lesson was that healthy living can help one “look healthy, feel good, and be happy”. Following the lesson and classroom discussion about healthy living, students were given a worksheet depicting numerous scenes portraying healthy and unhealthy habits. Students were instructed to identify five healthy habits and five unhealthy habits from the picture. The scenes were culturally relevant to the Indian lifestyle, as they depicted people eating typical Indian fast foods like pav-bhaji and chaatitems, drinking tender coconut water, and practicing yoga. Then, students were asked to identify three healthy and three unhealthy habits from their own lives. Student responses were checked for correctness and analyzed for pertinent themes.

### Appendix A.2. Activity 2

The second activity, *Climbing the Health Ladder*, introduces students to diabetes, risk factors, warning signs, and strategies to prevent or control the disorder. Following this lesson and classroom discussion, students were divided into small groups to play ‘snakes and ladders,’ a popular Indian game among children. In this game, snakes were labeled with risk factors while ladders were healthy lifestyle habits. All the students were given game sets to play with their families at home. No data were taken from this worksheet for analysis.

### Appendix A.3. Activity 3

The third activity, *Pass it On-The Traffic Signal*, educates students about healthy nutrition and balanced diets. They learn about food groups, serving sizes, empty calories, importance of breakfast, and methods of cooking. A three-zone food categorization was also introduced, with red (avoid completely or eat in very limited quantities), yellow (eat in moderation), and green (eat regularly) foods. The worksheet for this activity required students to design their own recipe, identify ingredients and method of cooking, and rate their recipe as red, yellow, or green. A major learning outcome from this activity is an understanding of how different cooking methods and selection of ingredients can affect the healthfulness of a meal.

### Appendix A.4. Activity 4

*Jump Forward and Race with Pace*, the fourth intervention activity, emphasizes the importance of physical activity in providing health benefits and reducing the risk of disease. The lesson introduces minimum physical activity levels for individuals of different age groups, and easy ways to incorporate physical activity into daily life. Obesity and its risk factors, complications, and preventative measures are also explained, along with information about BMI. Instead of a worksheet for this activity, students compete in a racing game with and without their backpacks. The learning outcome from this activity is for students to understand they can run faster and prevent diabetes when they do not have excess weight. A brief discussion ensued on the groups’ experience after undertaking the activity. Certificates were issued to the top three winners of the race. No objective data was needed from this activity for analysis, as the learning objective was self-evident once they completed the task.

### Appendix A.5. Activity 5

The final activity, *Doctor Says* “*Be Fit, Eat Right*” is a case study about an 11-year-old boy named Sachin and his lifestyle habits. Students were asked to identify healthy (green), moderately healthy (yellow), and unhealthy (red) habits from Sachin’s life, and then provide suggestions for small lifestyle modifications. The puzzle accompanying the case study reads, “Be Fit, Eat Right, Give Diabetes a Tough Fight”. The learning objective of this final activity is to recognize the importance of small lifestyle modifications to prevent the onset of type 2 diabetes.

## Appendix B

**Table A1:** Anthropometric measures of ORANGE study participants pre- and post-intervention, overall and by gender (*n* = 2276).

	Pre-Intervention	Post-Intervention	*p*-Value *
**Overall**	144.0 ± 8.2	147.0 ± 8.5	**<0.0001**
**Height**			
Boy	143.1 ± 8.2	146.1 ± 8.7	**<0.0001**
Girl	145.2 ± 8.1	148.1 ± 8.0	**<0.0001**
**Weight**			
Boy	36.3 ± 10.3	38.9 ± 10.9	**<0.0001**
Girl	38.3 ± 10.3	41.3 ± 10.8	**<0.0001**

* Difference was obtained by paired *t*-test.
